# Effects of Resistance Training in Youth Athletes on Muscular Fitness and Athletic Performance: A Conceptual Model for Long-Term Athlete Development

**DOI:** 10.3389/fphys.2016.00164

**Published:** 2016-05-09

**Authors:** Urs Granacher, Melanie Lesinski, Dirk Büsch, Thomas Muehlbauer, Olaf Prieske, Christian Puta, Albert Gollhofer, David G. Behm

**Affiliations:** ^1^Division of Training and Movement Sciences, Research Focus Cognition Sciences, University of PotsdamPotsdam, Germany; ^2^Department of Game and Combat Sports, Institute for Applied Training ScienceLeipzig, Germany; ^3^Department of Sports Medicine and Health Promotion, Friedrich-Schiller-University JenaJena, Germany; ^4^Department of Sport and Sport Science, Albert-Ludwigs-University FreiburgFreiburg, Germany; ^5^School of Human Kinetics and Recreation, Memorial University of NewfoundlandSt. John's, NL, Canada

**Keywords:** weight lifting, children, adolescents, physical fitness, muscle strength, muscle power, muscular endurance

## Abstract

During the stages of long-term athlete development (LTAD), resistance training (RT) is an important means for (i) stimulating athletic development, (ii) tolerating the demands of long-term training and competition, and (iii) inducing long-term health promoting effects that are robust over time and track into adulthood. However, there is a gap in the literature with regards to optimal RT methods during LTAD and how RT is linked to biological age. Thus, the aims of this scoping review were (i) to describe and discuss the effects of RT on muscular fitness and athletic performance in youth athletes, (ii) to introduce a conceptual model on how to appropriately implement different types of RT within LTAD stages, and (iii) to identify research gaps from the existing literature by deducing implications for future research. In general, RT produced small-to-moderate effects on muscular fitness and athletic performance in youth athletes with muscular strength showing the largest improvement. Free weight, complex, and plyometric training appear to be well-suited to improve muscular fitness and athletic performance. In addition, balance training appears to be an important preparatory (facilitating) training program during all stages of LTAD but particularly during the early stages. As youth athletes become more mature, specificity, and intensity of RT methods increase. This scoping review identified research gaps that are summarized in the following and that should be addressed in future studies: (i) to elucidate the influence of gender and biological age on the adaptive potential following RT in youth athletes (especially in females), (ii) to describe RT protocols in more detail (i.e., always report stress and strain-based parameters), and (iii) to examine neuromuscular and tendomuscular adaptations following RT in youth athletes.

## Introduction

**Figure 1:**
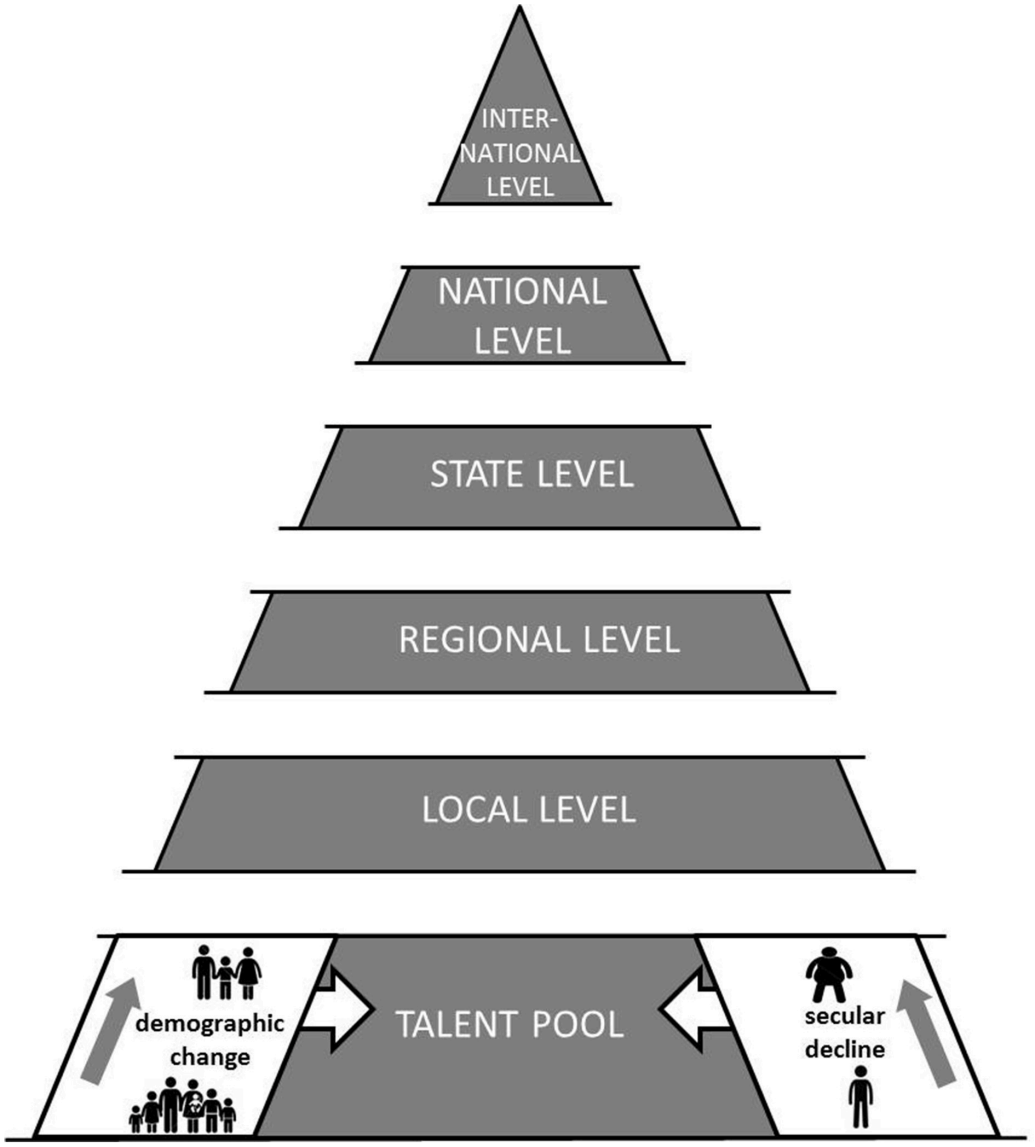
**Impact of demographic change and secular declines in motor performance on the pool of young talents with athletic potential to be introduced to long-term athlete development**.

The pool of youth with athletic potential to be introduced to long-term athlete development (LTAD) has become smaller in western industrialized countries due to demographic change and secular declines in motor performance (Figure 1). In 1950 for instance, 30% of Germany's population was under 20 years of age. An almost linear decline occurred over the following decades so that in 2013, only 18% of the German population was 20 years and younger (German Federal Statistical Office, 2015). In addition to demographic change, secular declines in youth motor performance were reported. These findings are not limited to Germany. Between 1981 and 2000, a meta-analysis revealed rapid performance declines in aerobic endurance (i.e., 0.43% per year) of children and adolescents aged 6–19 years living in developed countries (Tomkinson et al., 2003). Performance deteriorations were most marked in the older age groups but similar for boys and girls (Tomkinson et al., 2003). Secular declines were not only reported for aerobic endurance but also for muscular fitness (Runhaar et al., 2010). In this context, “muscular fitness” is used as an umbrella term for “muscular strength,” “local muscular endurance,” and “muscular power” (Smith et al., 2014). In Dutch children aged 9–12 years, Runhaar et al. (2010) observed a secular trend in muscular endurance (i.e., bent-arm hang) over a 26-years period ranging from −16 to −49%. This finding was supported by Cohen et al. (2011) who reported 10-years secular changes in measures of muscular fitness (i.e., hand grip strength [−6%], sit-ups [−27%], bent-arm hang [−26%]) in English children aged 10–11 years. From a health-related perspective, these declines are concerning because findings from a meta-analysis indicate an inverse association between muscular fitness and total and central adiposity, cardiovascular disease, and metabolic risk factors in youth (Smith et al., 2014). Further, positive associations were observed between muscular fitness and bone health and self-esteem (Smith et al., 2014).

Besides its function as a marker of health, muscular fitness is an essential component of athletic performance, which is why it plays an important role during the stages of LTAD. LTAD is a structured pathway to optimize the development from talented children into elite athletes that consists of seven sequential stages (*1. Active Start, 2. FUNdamentals, 3. Learn to Train, 4. Train to Train, 5. Train to Compete, 6 Train to Win, 7. Active for Life*) and considers individual maturational level rather than chronological age (Balyi et al., 2013). According to Ford et al. (2011), Lloyd et al. (2015), and Faigenbaum et al. (2016), muscular fitness should specifically be promoted at all stages of LTAD to support motor skill acquisition, to enhance motor performance, to improve markers of health and well-being, and to reduce the risk of sustaining sports-related injuries. Thus, youth athletes may benefit in three ways from implementing muscular fitness enhancing exercises in their regular training routine. First, by stimulating their athletic development/career, second, by tolerating the demands of long-term training and competition, and third, by inducing long-term health promoting effects that are robust over time and track into adulthood (stage 7 of LTAD) (Lloyd et al., 2015; Faigenbaum et al., 2016). The positive effects of resistance training (RT) on proxies of muscular fitness, health, sport-related, and everyday activities have been examined and described in healthy (non-athletic) children and adolescents in the form of randomized controlled trials (Granacher et al., 2014), systematic reviews (Benson et al., 2008), meta-analyses (Behringer et al., 2010), and position stands (Behm et al., 2008). However, findings from these studies can only partially be translated to the athletic context because physiology and proficiency in motor performance differ markedly between non-athletic and athletic youth (Armstrong and McManus, 2011). In other words, specific characteristics of youth athletes' physiology and level of expertise have an impact on their trainability (Lloyd et al., 2015). In addition, there is a gap in the literature with regards to optimal RT methods during LTAD and how RT is linked to biological age.

Thus, the aims of this scoping review were to describe and discuss the effects of RT on measures of muscular fitness and athletic performance in youth athletes (Table 1). In addition, a conceptual model will be introduced on how to appropriately implement different types of RT during the stages of LTAD (Table 2). Finally and in accordance with the principles of scoping reviews, we aimed at identifying research gaps in the literature and provided recommendations for future research (Table 3). Whereas prior reviews have described LTAD stages, others have summarized pediatric training-induced physiological adaptations; the present review attempts to integrate and apply training responses, mechanisms, and prescription through the stages of LTAD. A specific strength of this scoping review is that only studies examining youth athletes were included while previous work reported findings from athletic and non-athletic populations.

**Table 1:** **Summary of studies that were included in this scoping review on the effects of resistance training on muscular fitness and athletic performance in youth athletes**.

**Author**	**Characteristics of participants**						**Resistance training protocol**			**Outcomes**	
	**N**	**Biological age**	**Chronological age (years)**	**LTAD stage**	**Sex**	**Sport**	**Type of resistance training**	**Duration (weeks)**	**Frequency (sessions/week)**	**Comparator**	**Between-subject ES**
**MUSCULAR STRENGTH**											
Chelly et al., 2009	EG: 11; ACG: 11	n/a	EG: 17 ± 0.3; ACG: 17 ± 0.5	Training to train	M	Soccer	FW	8	2	EG vs. ACG	Half back squat (1 RM): ES = 1.74
Klusemann et al., 2012	EG I: 13; EG II: 11; ACG: 12	n/a	M: 14 ± 1; F: 15 ± 1	Training to train	M and F	Basketball	EG I: FT (supervised)	6	2	EG I vs. ACG	Push-up test: ES = 0.55
											Pull-up test: ES = 0.32
							EG II: FT (video-based)	6	2	EG II vs. ACG	Push-up test: ES = 0.54
											Pull-up test: ES = 0.32
Sander et al., 2013	EG I: 13; EG II: 30; EG III: 18 ACG I: 15; ACG II: 25; ACG III: 33	n/a	EG and ACG I: 17; EG and ACG II: 15; EG and ACG III: 13	Learning to train and training to train	n/a	Soccer	EG I: FW	80	2	EG I vs. ACG	Back squat (1 RM): ES = 2.96
											Front squat (1 RM): ES = 4.00
							EG II: FW	80	2	EG II vs. ACG	Back squat (1 RM): ES = 3.50
											Front squat (1RM): ES = 3.82
							EG III: FW	80	2	EG III vs. ACG	Back squat (1 RM): ES = 3.58
											Front squat (1RM): ES = 4.45
**MUSCULAR POWER**											
Hammami et al., 2016	EG I: 12; EG II: 12	Years from predicted PHV (EG I: –0.7 ± 0.3; EG II: –0.9 ± 0.4)	EG I: 12.7 ± 0.3; EG II: 12.5 ± 0.3	Learning to train	M	Soccer	EG I: BT + PT	8	2	EG I vs. EG II; (“+” in favor of EG I; “-” in favor of EG II)	Reactive strength index: ES = 2.04
							EG II: PT + BT	8	2		
											Absolute leg stiffness: ES = 1.56
											Relative leg stiffness: ES = 1.98
											Triple hop test: ES = 2.07
											Y balance test: ES = 1.38
Meylan and Malatesta, 2009	EG: 14; ACG: 11	n/a	EG: 13.3 ± 0.6; ACG: 13.1 ± 0.6	Learning to train	M	Soccer	PT	8	2	EG vs. ACG	CMJ: ES = 2.04
											SJ: ES = 1.29
											Reactive strength index: ES = 0.03
											Multiple 5 bound test: ES = 1.13
Ramírez-Campillo et al., 2014a	EG I: 13; EG II: 13; EG III: 11; ACG: 14	Prepubertal assessed through Tanner stage	10.4 ± 2.3	Learning to train	M	Soccer	EG I: PT (30 s inter-set rest)	7	2	EG I vs. ACG	CMJ: ES = 0.40
											Reactive strength index (20 cm): ES = 0.87
											Reactive strength index (40 cm): ES = 0.73
							EG II: PT (60 s inter-set rest)	7	2	EG II vs. ACG	CMJ: ES = 0.48
											Reactive strength index (20 cm): ES = 0.78
											Reactive strength index (40 cm): ES = 0.69
							EG III: PT (90 s inter-set rest)	7	2	EG III vs. ACG	CMJ: ES = 0.31
											Reactive strength index (20 cm): ES = 0.66
											Reactive strength index (40 cm): ES = 0.86
Santos and Janeira, 2011	EG: 14; ACG: 10	(Post-) pubertal assessed through Tanner stage	EG: 15.0 ± 0.5; ACG: 14.5 ± 0.4	Training to train	M	Basketball	PT	10	2	EG vs. ACG	CMJ: ES = 1.28
											SJ: ES = 2.02
											Abalakov test: ES = 1.36
											Depth jump: ES = 1.50
											Mechanical power: ES = 0.45
**MUSCULAR ENDURANCE**											
Christou et al., 2006	EG: 9; ACG: 9	(Post-) pubertal assessed through Tanner stage	EG: 13.8 ± 0.4; ACG: 13.5 ± 0.9	Training to train	M	Soccer	MB and FW	16	2	EG vs. ACG	30-s repeated jump test: ES = 0.27
DeRenne et al., 1996	EG I: 7; EG II: 8; ACG: 6	n/a	13.3 ± 1.3	Learning to train	M	Baseball	EG I: MB and FW	12	1	EG I vs. ACG	Pull-up test: ES = 1.06
							EG II: MB and FW	12	2	EG II vs. ACG	Pull-up test: ES = 1.33
Granacher et al., 2014	EG I: 13; EG II:14	n/a	EG I: 13.7 ± 0.6; EG II: 13.8 ± 0.	Training to train	M and F	n/a	EG I: FT on stable surfaces	6	2	EG I vs. EG II; (“+” in favor of EG I; “−” in favor of EG II)	Ventral TMS test: ES = −0.24
							EG II: FT on unstable surfaces	6	2		
											Dorsal TMS test: ES = 1.49
											Lateral right side TMS test: ES = 0.04
											Lateral left side TMS test: ES = 0.00
Weston et al., 2015	EG: 10; ACG: 10	n/a	EG: 15.7 ± 1.2; ACG: 16.7 ± 0.9	Training to train	M and F	Swimming	FT	12	3	EG vs. ACG	Prone bridge test: ES = 0.11
**ATHLETIC PERFORMANCE**											
Behringer et al., 2013	EG I: 12; EG II: 12; ACG: 12	(Post-) pubertal assessed through Tanner stage	15.0 ± 1.6	Training to train	M	Tennis	MB	8	2	EG I vs. ACG	Tennis service velocity: ES = 0.04
											Tennis service precision test: ES = 0.69
							PT	8	2	EG II vs. ACG	Tennis service velocity: ES = 1.39
											Tennis service precision test: ES = 0.51
Prieske et al., 2016a	EG I: 19; EG II: 18	n/a	EG I: 16.6 ± 1.1; EG II: 16.6 ± 1.0	Learning to train	M	Soccer	EG I: FT on stable surfaces	9	2–3	EG I vs. EG II; (“+” in favor of EG I; “–” in favor of EG II)	Kicking velocity: ES = −0.65
							EG II: FT on unstable surfaces	9	2–3		
Ramírez-Campillo et al., 2014b	EG: 38; ACG: 38	(Post-) pubertal assessed through Tanner stage	13.2 ± 1.8	Training to train	M	Soccer	PT	7	n/a	EG vs. ACG	Kicking test: ES = 0.88
Saeterbakken et al., 2011	EG: 14; ACG: 10	n/a	16.6 ± 0.3	Training to train	F	Handball	FT	6	2	EG vs. ACG	Throwing velocity: ES = 1.43

*Legend: ACG, active control group; BT, balance training; CMJ, countermovement jump; EG, experimental group; ES, effect size; F, female; FT, functional training (training with own body mass, thera-bands etc.), FW, free weights, LTAD, long term athlete development; M, male, MB, machine based; n/a, not applicable; N, number of subjects; PHV, peak height velocity; PT, plyometric training; RM, repetition maximum; SJ, squat jump; TMS, trunk muscle strength*.

**Table 2:** **Conceptual model for the implementation of resistance training (RT) programs during the stages of long-term athlete development (LTDA) to enhance muscular fitness and athletic performance**.

**Early childhood**	**Late childhood**	**Adolescents**	**Adulthood**
**CHRONOLOGICAL AGE**			
Female: 6–8 years	Female: 9–11 years	Female: 12–18 years	Female: >18 years
Male: 6–9 years	Male: 10–13 years	Male: 14–18 years	Male: >18 years
**BIOLOGICAL AGE**			
Tanner stage I	Tanner stage I–II	Tanner stage III–IV	Tanner stage V
**MATURITY**			
Pre-pubertal (pre PHV)	Pre-pubertal (pre PHV)	Pubertal (mid PHV)	Post-pubertal (post PHV)
**STAGE OF LONG-TERM ATHLETE DEVELOPMENT**			
FUNdamentals	Learning to train	Training to train	Training to compete
**LONG-TERM DEVELOPMENT OF MUSCULAR FITNESS (STRENGTH, POWER, ENDURANCE)**			
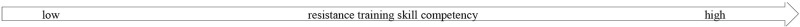			
- Coordination training - Agility training - Balance training - Muscular endurance training with own body mass/training tools (e.g., medicine ball) with a focus on exercise technique	- Balance training - Plyometric training as part of deliberate play (e.g., rope skipping) with a focus on correct jumping and landing mechanics - Core strength training - Muscular endurance training with own body mass/training tools (e.g., medicine ball) - Free weight training with a focus on exercise technique	- Balance training - Plyometric training (depth jumps from low drop heights) - Core strength training - Free weight training at light to moderate loads - Heavy resistance strength training (hypertrophy) - Eccentric resistance training - Sport-specific resistance training	- Balance training - Plyometric training (depth jumps from moderate drop heights) - Core strength training - Free weight training at moderate to high loads - Heavy resistance strength training (neuromuscular activation + hypertrophy) - Sport-specific resistance training
**TRAINING-INDUCED ADAPTATIONS**			
Neuronal adaptations		Hormonal/Neuronal/Muscular/Tendinous adaptations	

*RT programs were allocated to LTAD stages based on expert opinion and according to Lesinski et al. (2016), Faigenbaum et al. (2016), Lloyd et al. (2011, 2015), Balyi et al. (2013), as well as Kraemer and Fleck (2005)*.

*Legend: PHV, peak height velocity*.

**Table 3:** **Identified research gaps in the literature and recommendations for future studies**.

**Identified problems from scoping review**	**Implications for future research**
Lack of studies that examined RT effects in child athletes	Particularly examine the effects of RT in child athletes
Lack of studies that reported measures of biological age	Always determine and report a measure of biological age (e.g., peak-height velocity)
Lack of studies that examined sex-specific effects of RT	Particularly examine the effects of RT in female youth athletes
Lack of studies that examined physiological adaptive processes following RT in child and adolescent athletes	Elucidate neuromuscular and tendomuscular mechanisms following RT in youth athletes according to sex and biological age
Insufficient reporting and inclusion of stress and strain-based parameters in RT studies	Describe RT protocols in more detail (report stress and strain-based parameters)
Insufficient matching of RT protocols when comparing different protocols	When comparing different RT protocols make sure that protocols are matched for strain-based parameters (e.g., time under tension) or mechanical work (i.e., lifted overall load)

*Legend: RT, resistance training*.

Of note and in accordance with Behm et al. (2008), we define RT as a specialized method of conditioning that involves the progressive use of a wide range of resistive loads, including body mass, and a variety of training modalities (e.g., machine-based training, free weight training, plyometric training, complex training, functional training) designed to enhance muscular fitness and athletic performance.

## Methods

This article is presented in the form of a scoping review (Armstrong et al., 2011). According to the principles of a scoping review the research question was broad. For instance, “Can RT increase muscular fitness and athletic performance in youth athletes?” or “What are appropriate and evidence-based RT methods to be implemented during the stages of LTAD?.” Typically, a scoping review can be used to identify research gaps in the existing literature (Table 3) as well as to summarize research findings (Table 1) without necessarily performing a study quality assessment or an extensive data synthesis in the form of a systematic review and meta-analysis (Armstrong et al., 2011). We identified relevant studies listed in review articles (Lloyd et al., 2014, 2015; Faigenbaum et al., 2016; Lesinski et al., 2016) with the key inclusion criterion that the subjects were youth sub-elite, elite, and/or competitive athletes. According to Araujo and Scharhag (2016), the term “athletes” was defined as a person to be training in sports aiming to improve performance. We additionally checked the reference lists of each included article in an effort to identify additional suitable studies for inclusion. Studies were not included if they failed to specify the athletic level. Finally, all included studies had to examine the effects of RT (≥6 training sessions) on at least one measure of muscular fitness (i.e., strength, power, endurance) and/or athletic performance (i.e., proxies of performance in specific sport disciplines). Whenever possible, effect sizes (ES) were reported for the included studies to illustrate the practical relevance of the respective study outcomes. Between-subject standardized mean differences (corresponds to ES) were computed according to the following formula: standardized mean differences = mean post value intervention group-mean post value control group)/pooled standard deviation). We adjusted the standardized mean differences for the respective sample size by using the term (1−(3/(4N-9))). According to Rhea's (2004) scale for determining the magnitude of ES in strength training research for individuals who have been consistently training for 1–5 years (i.e., youth athletes), we interpreted ES as trivial (<0.35), small (0.35–0.79), moderate (0.80–1.50), or large (≥1.50).

## Effects of resistance training on muscular fitness in youth athletes

To the authors' knowledge, there are three meta-analyses available in the literature which examined the effects of RT on measures of muscular fitness in healthy (non-athletic) youth. These three studies (Falk and Tenenbaum, 1996; Payne et al., 1997; Behringer et al., 2010) clearly illustrate the general effectiveness of RT to improve muscular fitness in youth. The reported ES ranged from trivial (ES = 0.20) to large effects (ES = 1.91) depending on biological/chronological age, sex, and mode of muscle action (i.e., isometric, isotonic, isokinetic protocols). However, findings from these meta-analyses cannot directly be transferred to youth athletes because athletes differ from healthy but untrained youth with regards to training capacity, adherence, physical demands of activities, physical condition, and injury risk (Bergeron et al., 2015). Thus, it is imperative to report cohort specific results when discussing and interpreting the effects of RT in youth athletes which will be done in the following.

### Effects of resistance training on muscular strength in youth athletes

Muscular strength can be defined as the maximal force or tension a muscle or a group of muscles can generate at a specified velocity (Knuttgen and Kraemer, 1987). Many sport-specific situations demand high accelerations of an external resistance (e.g., own body mass, body mass of opponent, mass of object) for athletic success in competition. According to Newton's second law of motion (Force = mass • acceleration), the acceleration of an external resistance is determined by the magnitude of an acting force. This clearly illustrates that the ability to voluntarily produce a maximal force or torque is important for sports performance. In addition, Schmidtbleicher (2004) considered maximal muscular strength as the basic dimension of muscular fitness. In other words, proficiency level of maximal muscular strength influences performance in muscular power and muscular endurance. This implies that training-induced improvements in maximal muscular strength result in concomitant enhancements of muscular power and muscular endurance (Schmidtbleicher, 2004) which is why conditioning programs should focus on the development of maximal muscular strength.

A number of studies investigated the effects of RT on measures of maximal muscular strength in youth athletes (Chelly et al., 2009; Klusemann et al., 2012; Sander et al., 2013) (Table 1). A study that is of particular interest in this context was conducted by Sander et al. (2013) because it examined the effects of long-term strength training (i.e., 2 years) on measures of maximal strength of the lower extremities in three age groups (i.e., under 13, 15, and 17 years) of male elite soccer players. For this purpose, participants from each age group were allocated to a RT or an active control group. The RT group conducted four regular soccer training and two additional RT sessions per week. During RT, subjects performed parallel front and back squat, bench press, neck press, deadlift, and core exercises. A periodization model was applied for the squat exercises with a focus on technical skill training during the first 4 weeks. For the next 8 weeks, the focus emphasized inducing muscle hypertrophy with a training protocol consisting of five sets, 10 repetition maximum (RM), and 3 min rest between sets. During the next 4 weeks, training intensity was increased to five sets, six RM, and a 3 min rest between sets. The last 4 week cycle consisted of five sets, four RM, and a 5 min rest between sets. The RT protocol for the upper extremities and the core muscles comprised three to five sets, 10 RM, and a 3 min rest between sets over the course of the training period. This 20-week periodization model was conducted twice during one soccer season and four times over the 2 years intervention period. The active control group conducted regular soccer training over the course of the study with four soccer sessions per week and no specific RT. One RM front and back squat tests were performed on three occasions, prior to the start of the study, after 1 and 2 years. Findings revealed significant group by time interactions for the front and back squat 1 RM in favor of the three age-specific RT groups. The largest ES were observed for the youngest age group with ES = 1.9 for the front squat and ES = 2.0 for the back squat with tremendous relative percentage increases (% RT group minus % active control group) of 230 to 250% (front/back squat) over the 2 years training period. This relative increase was lower in the older two cohorts and ranged between 56 and 80% indicating that the RT induced adaptive potential was largest in those subjects that were under 13 years of age at the beginning of the study.

**Figure 2:**
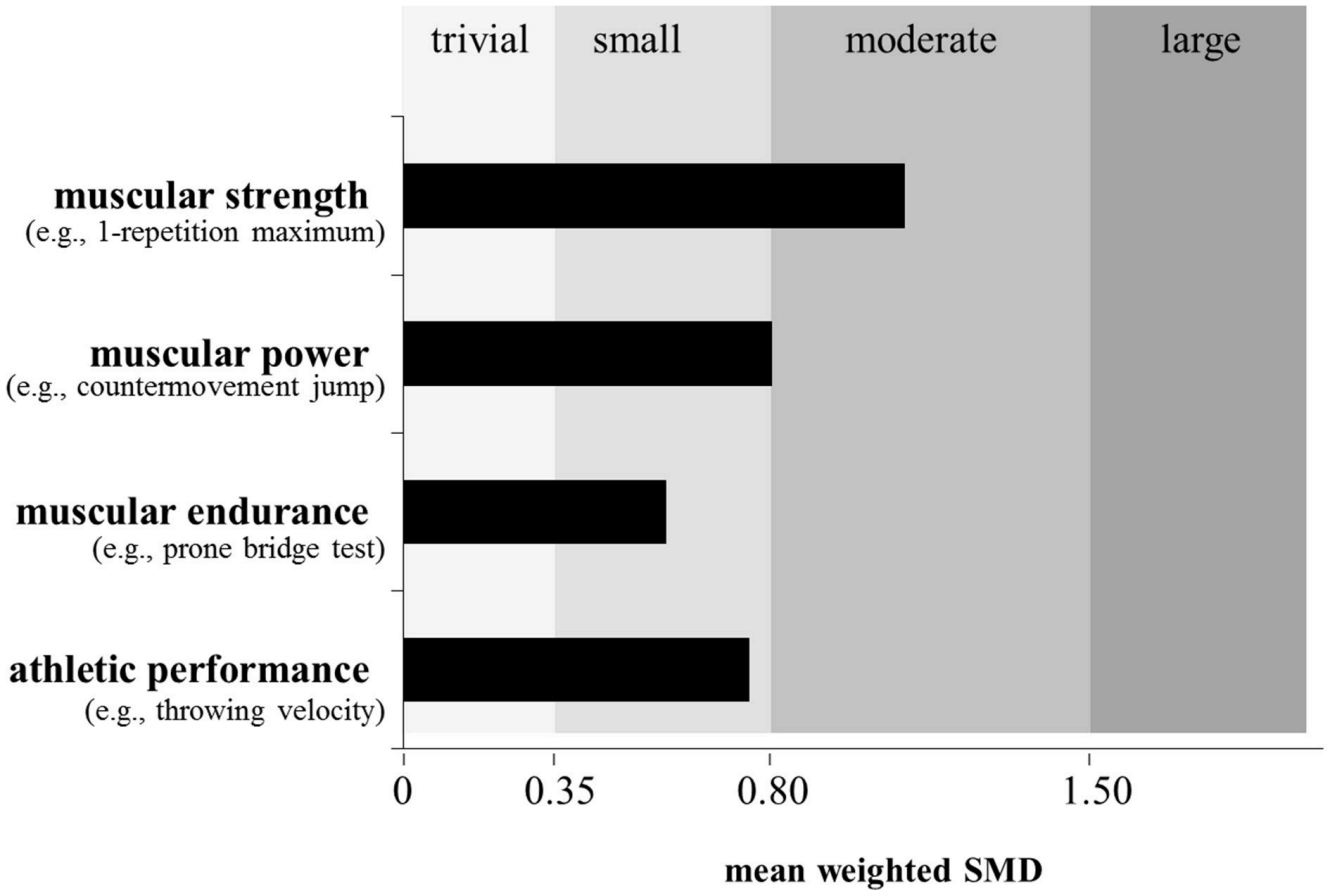
**Effects of resistance training on measures of muscular strength (*n* = 16 studies), muscular power (*n* = 33 studies), muscular endurance (*n* = 3 studies), and athletic performance (*n* = 20 studies) in youth athletes**. Of note, only studies with an active control group were included if they investigated the effects of resistance training in youth athletes (6–18 years) and tested at least one measure of muscular fitness and athletic performance. Legend: SMD = standard mean difference (effect size). Modified from Lesinski et al. (2016).

**Figure 3:**
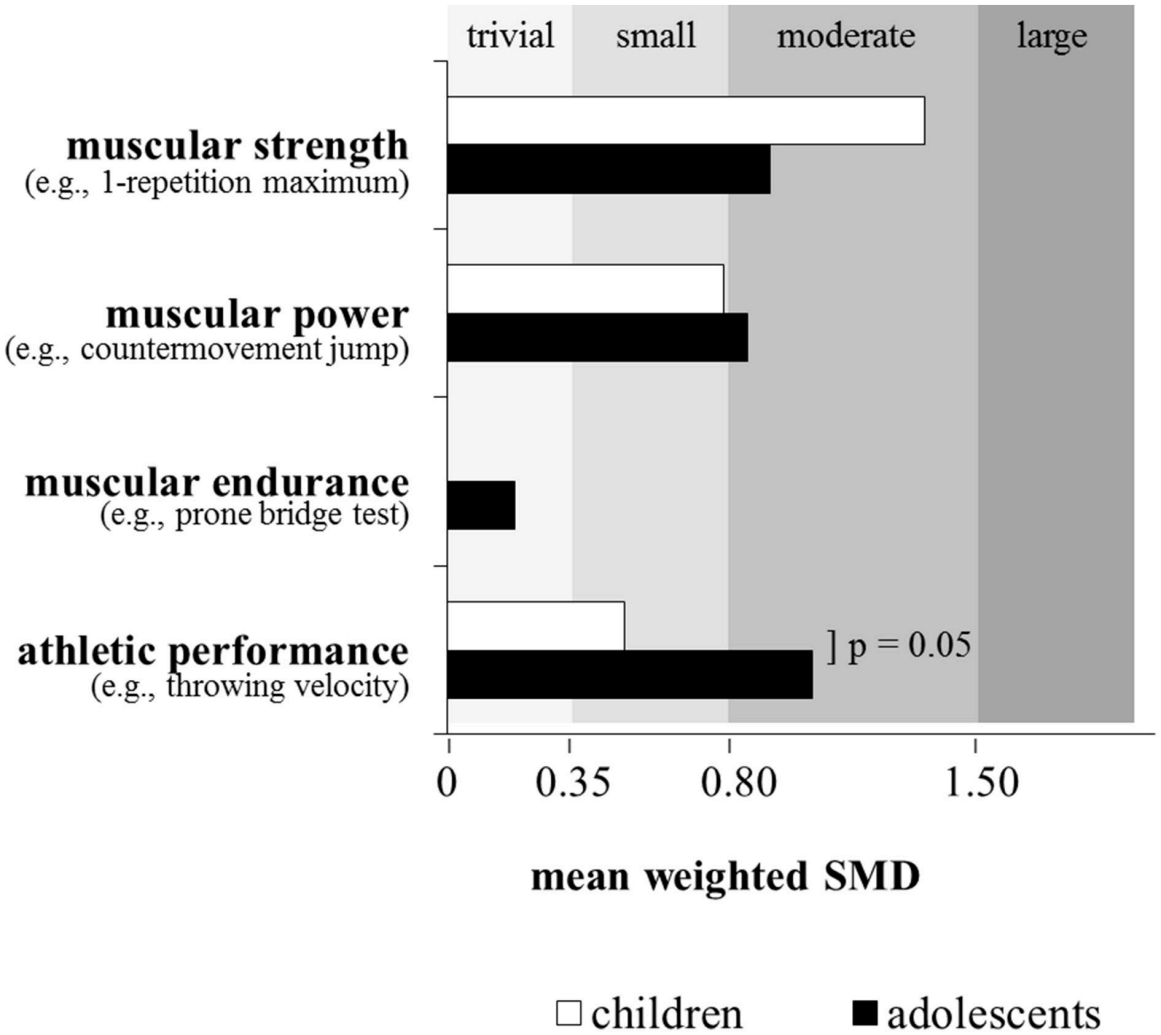
**Effects of resistance training on measures of muscular strength (children: *n* = 3 studies; adolescents: *n* = 13 studies), muscular power (children: *n* = 10 studies; adolescents: *n* = 22 studies), muscular endurance (adolescents: *n* = 2 studies), and athletic performance (children: *n* = 6 studies; adolescents: *n* = 13 studies) in youth athletes depending on chronological age**. Of note, only studies with an active control group were included if they investigated the effects of resistance training in youth athletes (6–18 years) and tested at least one measure of muscular fitness and athletic performance. Legend: *p* = *p*-value refers to the respective subgroup analysis; SMD = standard mean difference (effect size). Modified from Lesinski et al. (2016).

Lesinski et al. (2016) recently conducted a systematic review and meta-analysis on the effects of RT on measures of muscular fitness and physical performance in youth athletes aged 6 to 18 years. The systematic search revealed 43 studies that were eligible for inclusion in the meta-analysis. Based on findings from 16 studies, the analysis indicated moderate effects (ES = 1.09) of RT on measures of muscular strength (Figure 2). In addition, an age-group specific sub-analysis showed larger effects for children (boys ≤ 13 years; girls ≤ 11 years; ES = 1.35) as compared to adolescents (boys 14–18 years; girls 12–18 years; ES = 0.91), which strengthens the findings of Sander et al. (2013) (Figure 3). The larger adaptive potential following RT in child athletes can most likely be explained by greater relative (percentage) strength gains and increased neural plasticity in children as compared to adolescent athletes (Pfeiffer and Francis, 1986; Ramsay et al., 1990). Finally, the meta-analysis of Lesinski et al. (2016) revealed that free weight training produced the largest effects (ES = 2.97) on measures of muscular strength in youth athletes followed by the combination of machine-based and free weight training (ES = 1.16), functional training (ES = 0.62), plyometric training (ES = 0.39), and machine-based training (ES = 0.36) (Figure 5). The superiority of free weight training either in isolation or in combination can be attributed to the additional muscular stabilization of the trunk and limb joints needed to control the greater degrees of freedom associated with the multi-planar movements (Behm et al., 2010).

In summary, RT is an effective means to improve muscular strength in youth athletes of all ages, with the introduction of free weights RT from the late childhood LTAD stage and beyond (Table 2). It appears that trainability in terms of relative strength gains is higher in child athletes as compared to adolescent athletes and that free weight training is particularly effective. Further, it is recommended that researchers consistently report information on the maturation status of the investigated cohort (e.g., peak height velocity, Tanner stages) because chronological and biological age can largely differ in children and adolescents (Table 3).

### Effects of resistance training on muscular power in youth athletes

Muscular power refers to the rate at which muscles perform work (Power = work/time) (Smith et al., 2014). The importance of muscular power for the athletic context becomes clearly manifested when inserting the term work = force • distance in the equation Power = work/time resulting in the deduced term Power = force • velocity. In other words, dynamic muscle actions at high forces, and movement velocities are essential components of power production. Furthermore, the concept of the velocity specificity of RT indicates that the greatest increases in strength or power occur at or near the velocity of the training exercises (Behm and Sale, 1993). This implies that both components of the power equation (i.e., force and velocity) have to be trained if the goal is to maximize muscular power. In this context, we explicitly defined “power” in Newtonian mechanics and we comply with Winter et al. (2016) that the use of the term “power” in the exercise scientific literature should be limited to the true definition of mechanical power. Nevertheless, when conducting this literature review we were confronted with several studies that used a rather colloquial meaning of “power” as a surrogate for muscular performance that includes extremes of force or speed. We decided not to exclude those studies to get a comprehensive picture of the scientific literature. However, we recommend that future studies stick to the mechanical definition of the term “power.”

**Figure 4:**
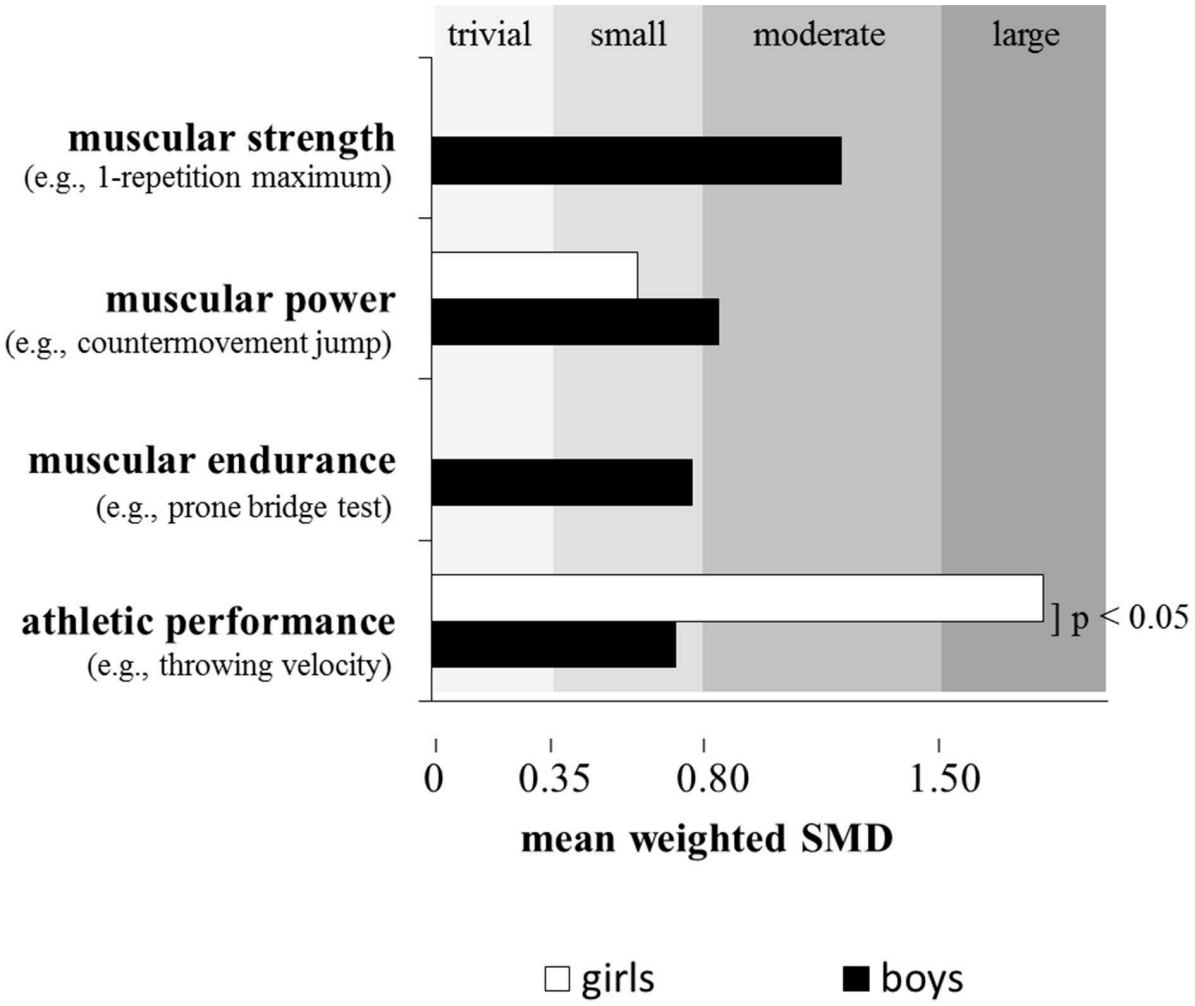
**Effects of resistance training on measures of muscular strength (boys: *n* = 12 studies), muscular power (girls: *n* = 3 studies; boys: *n* = 27 studies), muscular endurance (boys: *n* = 2 studies), and athletic performance (girls: *n* = 2 studies; boys: *n* = 15 studies) in youth athletes depending on sex**. Of note, only studies with an active control group were included if they investigated the effects of resistance training in youth athletes (6–18 years) and tested at least one measure of muscular fitness and athletic performance. Legend: *p* = *p*-value refers to the respective subgroup analysis; SMD = standard mean difference (effect size). Modified from Lesinski et al. (2016).

**Figure 5:**
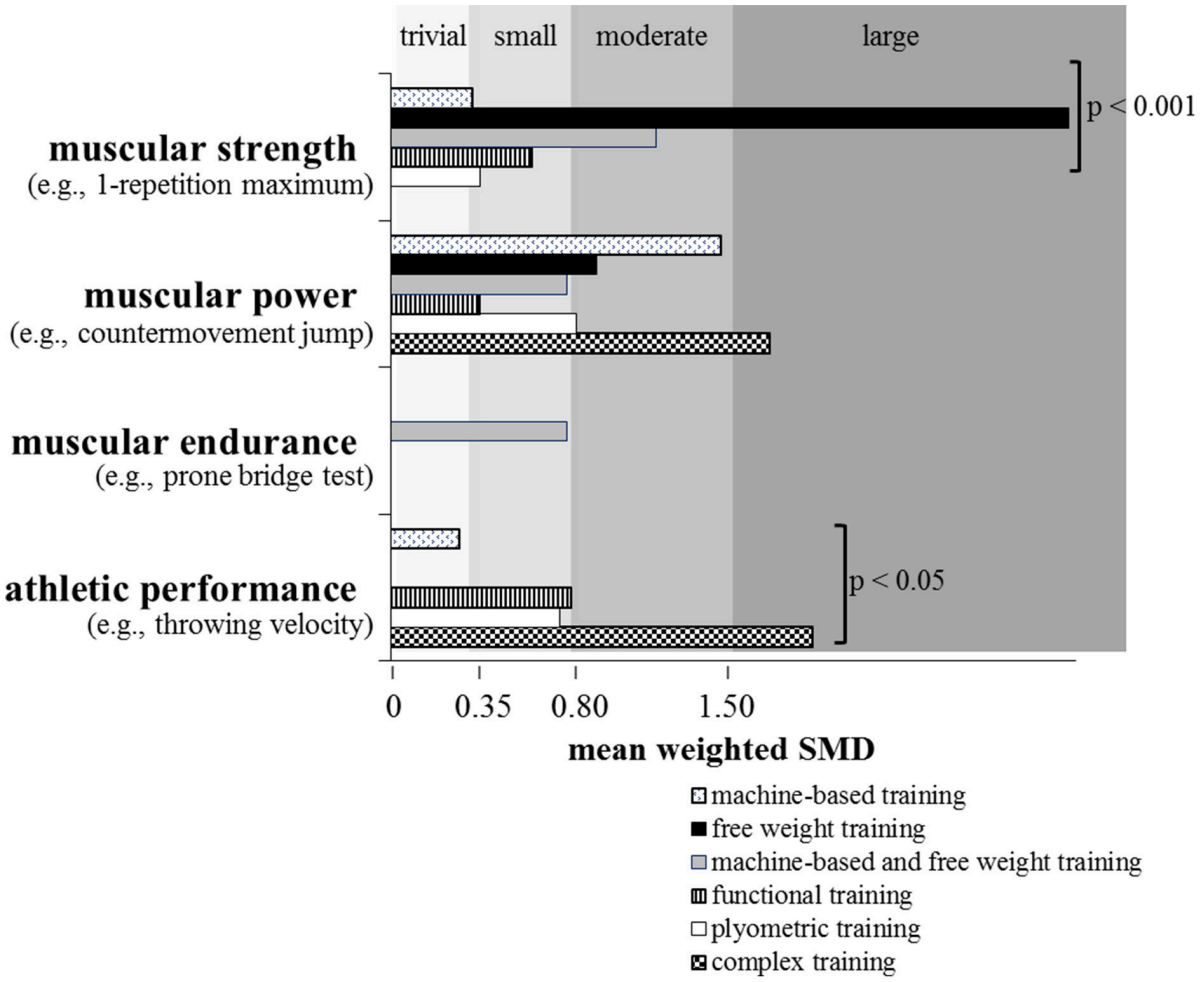
**Effects of resistance training on measures of muscular strength (machine-based training: *n* = 3 studies; free weight training: *n* = 2 studies; machine-based and free weight training: *n* = 4 studies; functional training: *n* = 2 studies; plyometric training: *n* = 4 studies), muscular power (machine-based training: *n* = 3 studies; free weight training: *n* = 3 studies; machine-based and free weight training: *n* = 3 studies; functional training: *n* = 2 studies; plyometric training: *n* = 16 studies; complex training: *n* = 4 studies), muscular endurance (machine-based and free weight training: *n* = 2 studies), and athletic performance (machine-based training: *n* = 3 studies; functional training: *n* = 5 studies; plyometric training: *n* = 10 studies; complex training: *n* = 2 studies) in youth athletes depending on type of resistance training**. Of note, only studies with an active control group were included if they investigated the effects of resistance training in youth athletes (6–18 years) and tested at least one measure of muscular fitness and athletic performance. Legend: *p* = *p*-value refers to the respective subgroup analysis; SMD = standard mean difference (effect size). Modified from Lesinski et al. (2016).

There is evidence in the literature that plyometric training is well-suited to enhance muscular power (de Villarreal et al., 2009) and/or proxies or surrogates of muscular power because plyometrics (e.g., hops, jumps) refer to exercises that link strength with speed of movement (Faigenbaum and Chu, 2001). More specifically, plyometric exercises start with a rapid stretch of a muscle or muscle group during the breaking or eccentric phase resulting in high forces at ground contact. During the subsequent push-off or concentric phase, the same muscle or muscle group shortens rapidly to accelerate the body in vertical direction. Thus, both components of the power equation are stimulated during plyometric training. Consequently, plyometric training is an often applied method during the stages of LTAD (Table 2) particularly in sports like soccer, basketball, and handball (Santos and Janeira, 2011; Ramírez-Campillo et al., 2014a) (Table 1). The general effects of in-season plyometric training on proxies of muscular power (i.e., countermovement jump [CMJ] height, hurdle jump height) were shown by Meylan and Malatesta (2009) for early pubertal soccer players with a mean age of 13 years. Following 8 weeks of training with two training sessions per week, significant improvements were found in the intervention as compared to the control group in CMJ (+7.9%) and hurdle jump height (+10.9%). These performance enhancements were substantiated by the already presented meta-analysis of Lesinski et al. (2016) who reported moderate effects (ES = 0.80) of RT on proxies of muscular power in youth athletes (Figure 2). This is in accordance with a previously conducted meta-analysis by Harries et al. (2012) who aggregated findings from 14 studies and reported similar effects of RT on proxies of muscular power in adolescent athletes. Lesinski et al. (2016) additionally examined age- and sex-specific effects of RT on muscular power and observed slightly smaller effect sizes for child (ES = 0.78) compared to adolescent athletes (ES = 0.85) (Figure 3) and larger effect sizes for boys (ES = 0.85) compared to girls (ES = 0.61) (Figure 4). The sub-analysis regarding training type revealed that complex training which combines weight and plyometric exercises during the same training session produced the largest effects (ES = 1.66) on proxies of muscular power in youth athletes followed by machine-based training (ES = 1.45), free weight training (ES = 0.90), plyometric training (ES = 0.81), the combination of machine-based, and free weight training (ES = 0.77), and functional training (ES = 0.39) (Lesinski et al., 2016) (Figure 5). The inability of plyometric training alone to provide the greatest magnitude of change may be ascribed to the greater balance challenges with this activity. Behm et al. (2010) in their review reported that unstable environments (bases and implements) tend to decrease force output compared to more stable environments. Hence, since balance capabilities are immature and not fully developed in youth (Behm et al., 2010), the Lesinski et al. (2016) findings emphasize the need for RT to be included with plyometric training for optimal power gains with youth.

Taken together, these findings clearly illustrate that both components of the power equation (i.e., force, velocity) have to be emphasized to induce large training-induced effects on muscle power. While the findings of the general effects of RT on muscular power in youth athletes are robust (Lesinski et al., 2016), the results of the age- and sex-specific RT effects (Lesinski et al., 2016) have to be interpreted cautiously due to a limited number of studies that were included in the meta-analysis (see captions of Figures 3, 4). Nevertheless, the larger effects in adolescent compared to child athletes and boys compared to girls could be explained by biological maturation (i.e. children vs. adolescents) and maturational differences (i.e., boys vs. girls). There is in fact evidence from magnetic resonance imaging (MRI) studies that muscle cross-sectional area increases with age from childhood through adolescence and more in boys compared to girls (De Ste Croix et al., 2002). Besides changes in muscle mass with maturation, there is knowledge of sex-specific fiber type growth and distribution particularly during adolescence. While percentage of type I fibers is equal in boys and girls during childhood, there are apparent differences during adolescence with females having a lower percentage of type I fibers than males and male type II fibers being bigger than their type I fibers which is not the case in females (Vogler and Bove, 1985; Glenmark et al., 1992). Given that muscle mass and fiber type distribution are important prerequisites for the generation of muscular power, the described age- and sex-specific physiological characteristics may partially explain the reported outcomes of Lesinski et al. (2016).

In summary, RT is an effective means to improve proxies of muscular power in youth athletes of all ages, with free weight RT and plyometric training best introduced at the late childhood stage (Table 2). Training types that emphasize both components of the power equation appear to produce the largest effects (i.e., complex training). The reported age- and sex-specific effects might be caused by methodological limitations (i.e., lack of studies in girls and child athletes) and/or maturational and sex-specific physiological characteristics (Table 3). Finally, more research is needed to elucidate the underlying neuromuscular and tendomuscular adaptations following RT in youth athletes and to separate training-induced adaptations from growth and maturation.

### Effects of resistance training on muscular endurance in youth athletes

Local muscular endurance is the ability to voluntarily generate consistent or repeated submaximal force or torque output (>30% 1 RM) utilizing a single muscle or a group of muscles for an extended period of time while resisting fatigue (Moir, 2012). If for instance the goal is to maintain high movement speed during an 800-m running competition or to minimize the fatigue-related performance decrements, the impulse-momentum theorem (force • time = mass • Δvelocity) illustrates that if body mass remains constant, the magnitude of the impulse (force • time) over the competition/race time is directly related to the achieved momentum (mass • Δvelocity), i.e., step velocity and ultimately race pace. Thus, adequate RT programs are needed in specific sports disciplines like rowing, running, swimming, kayaking and others, which promote the ability of the active musculature to resist fatigue and to optimize performance.

So far, only a few studies examined the specific effects of RT on measures of local muscular endurance in youth athletes (Granacher et al., 2014; Weston et al., 2015). A recently published randomized controlled trial (Granacher et al., 2014) studied the effects of a 6 week core RT using stable vs. unstable surfaces (e.g., Thera-band^©^ stability trainer) on local muscular endurance of the trunk in male and female youth athletes aged 14 ± 1 years. Subjects trained two times per week performing static and dynamic frontal, dorsal, and lateral core exercises (i.e., curl-up, side bridge, quadruped position) for three sets per exercise with progressively increasing contraction time (static condition: 40–50 s) and/or number of repetitions (dynamic condition: 20–25 repetitions). Following training, both intervention groups significantly improved local muscular endurance of the ventral and lateral trunk muscles in a range of 8–41%, independent of surface conditions. In addition, Weston et al. (2015) determined the effects of a 12-week core RT on local muscular endurance of the trunk (i.e., timed prone-bridge test) in national-level adolescent swimmers (16 ± 1 years) compared to an active control. The intervention group performed three training sessions per week, incorporating static and dynamic exercises (e.g., prone bridge, side bridge, bird dog etc.) with two to three sets per exercise and a progressively increasing contraction time (static condition: 30-90 s) and/or number of repetitions (dynamic condition: 10–30 repetitions). After training, the intervention group showed small improvements on the timed prone-bridge test compared to the active control (+9.0%).

To the authors' knowledge, there are only three studies (DeRenne et al., 1996; Christou et al., 2006; Weston et al., 2015) available that examined the effects of RT on measures of local muscular endurance in youth athletes compared to an active control (Table 1). Findings from these three studies were aggregated and presented in Figure 2. An ES of 0.57 is indicative of a small effect of RT on local muscular endurance measures with youth athletes (Figure 2). Because of the limited number of studies available in the literature, age-, sex-, and RT- type-specific sub-analyses were not applicable (Figures 3–5).

In summary, results from four studies indicate that youth athletes can improve their local muscular endurance following RT. However, the aggregated effects of RT on measures of local muscular endurance were small compared to the previously reported moderate effects of RT on measures of muscular strength and power (Figure 2). Considering the small number of available studies concerning this topic (Table 1), more research is needed to elucidate and substantiate the effects of RT on local muscular endurance in youth athletes.

## Effects of resistance training on athletic performance in youth athletes

There is evidence that RT has the potential to improve muscular strength, muscular power, muscular endurance, agility, balance and stability, coordination, and speed of movement in youth athletes (Harries et al., 2012; Lesinski et al., 2016). These training-induced gains in health and skill-related physical fitness parameters may support young athletes during the acquisition phase of complex movements, for mastering sport tactics, and to withstand the demands of long-term athletic training and competition (Faigenbaum et al., 2016). Given this broad spectrum of RT efficacy, it is plausible to argue that the previously reported effects of RT in youth athletes translate to their athletic performance (Faigenbaum et al., 2016). In this context, the term “athletic performance” refers to proxies of performance in specific sport disciplines for instance, soccer (e.g., kicking velocity), handball (e.g., throwing velocity), baseball (e.g., hitting and throwing velocity), tennis (e.g., velocity of tennis-serve), and swimming (e.g., start and swimming performance).

Saeterbakken et al. (2011) examined the effects of a 6-week, two times per week sling exercise RT on maximal throwing velocity among female handball players with a mean age of 16.6 ± 0.3 years (Table 1). After training, maximal throwing velocity significantly increased 4.9% in the intervention group but was unchanged in the control group. These results suggest that core stability training using unstable, closed kinetic chain movements can significantly improve maximal throwing velocity in youth female handball players. In another study, Ramírez-Campillo et al. (2014b) scrutinized the effectiveness of low-volume and high-intensity plyometric training on maximal kicking distance in youth soccer players with a mean age of 13.2 ± 1.8 years. After 7 weeks of training with two training sessions per week, maximal kicking distance significantly improved by 14% in the intervention compared to the control group. Further, Behringer et al. (2013) evaluated the transferability of two different RT protocols (i.e., upper/lower extremity plyometric training vs. upper/lower extremity machine-based RT) on service velocity and its precision consistency in junior tennis players with a mean age of 15.0 ± 1.6 years (Table 1). Following a training period of 8 weeks with two training sessions per week in addition to the regular tennis training, mean service velocity over 20 maximum-velocity serves increased significantly more in the plyometrics group (+3.8%) when compared with a control group. No significant changes were found in the machine-based RT group (+1.2%). Service precision (i.e., 20 targeted maximum-velocity serves from the baseline to the intercept point of the service line and the center service line) did not significantly change from pre- to post-test in all three experimental groups.

In their meta-analysis, Lesinski et al. (2016) aggregated results from 20 studies and found overall small effects of RT on proxies of athletic performance in youth athletes (ES = 0.75) (Figure 2). Age- and sex-specific sub-analyses revealed a tendency toward significantly larger effects in adolescent athletes (ES = 1.03) compared to children (ES = 0.50) (Figure 3) and significantly larger effects for girls (ES = 1.81) compared to boys (ES = 0.72) (Figure 4). In terms of training type, larger effects were found for complex training (ES = 1.85) followed by functional training (ES = 0.79), plyometric training (ES = 0.74), and machine-based training (ES = 0.30) (Figure 5). As most athletic performances demand a power component, it is consistent that similar to the findings on the effect of training type on muscular power that the complex training involving both plyometrics and RT demonstrated the greatest training gains.

Again, it is highly speculative to interpret age- and sex-specific effects of RT on proxies of athletic performance in youth athletes. Lesinski et al. (2016) aggregated six studies to illustrate the specific effects of RT in children while 13 studies were summarized in adolescents. Only two studies examined sex-specific RT effects in girls while 15 studies scrutinized this subject in boys (Lesinski et al., 2016). Therefore, methodological issues might account for the observed findings. In addition, physiological reasons may also explain the larger adaptive potential in adolescents compared to children because adolescents have larger muscle mass and type II fibers compared to children (Vogler and Bove, 1985; Glenmark et al., 1992), which may account for the higher trainability of athletic performance in adolescents. Finally, female athletes may possess larger adaptive reserves than their male counterparts to enhance athletic performance due to a larger potential to respond to neural stimuli (Streckis et al., 2007).

In summary, RT produces only small effects on proxies of athletic performance in youth athletes. Among the examined training types, complex training appears to be the best-suited agent to improve athletic performance. The reported age- and sex-specific effects could be due to methodological limitations (i.e., lack of studies in girls and children) and/or maturational and sex-specific physiological characteristics (Table 3).

## Practical implications for coaches and fitness professionals

It has been established for years by a number of national associations (i.e., American, Australian, British, Canadian, German and others) that RT is an acceptable and safe mode of training for children and adolescents with positive effects on health, psycho-social skills, well-being, and a reduction in the severity and incidence of injuries (Behm et al., 2008; Lloyd et al., 2014). In regards to older myths, misinformation regarding the potential negative effects of RT for children has been refuted, and thus coaches, fitness professionals, and young athletes can focus on the optimal training regimens to enhance muscular fitness and athletic performance.

If a primary characteristic of the sport necessitates the development of muscular strength, the present findings show that it is most optimally improved with the use of free weight resistance exercises. Free weights (e.g., dumbbells, barbells) permit multi-planar movements around the three axes through a spectrum of velocities, which allow a greater diversity of more task and velocity specific resisted actions. This freedom of movement also places greater stress on the maintenance of balance and stability, which are requisites for almost all athletic endeavors. Since, balance and coordination are not fully matured in children (Payne and Isaacs, 2008), balance training would be an important preliminary training phase for enhancing latter strength and power training progressions and reducing the risk of athletic and RT injury (McGuine and Keene, 2006). Balance is essential for optimal athletic performance (McGuine and Keene, 2006). Hence, balance training should be incorporated prior to and during strength and power training (Table 2). In accordance with this recommendation, Hammami et al. (2016) trained young elite soccer players aged 12–13 years twice per week for 8 weeks either with an initial 4 weeks of balance training followed by 4 weeks of plyometric training or 4 weeks of plyometric training proceeded by 4 weeks of balance training. Balance training prior to plyometric training initiated greater training improvements in reactive strength, leg stiffness, triple hop test, and the Y balance test. Furthermore, Chaouachi et al. (2014) trained 12–15 years old boys over an 8-week training period with either plyometric exercises only or with a combination of balance and plyometric training. There were no differences in 8 of the 11 balance and power measures. However, with only half the volume of plyometric training, the combined training enhanced leg stiffness, 10-m sprints and shuttle runs to a greater degree. Thus, balance exercises should play a significant prior and concurrent role in the strength and power training of child and adolescent athletes, which was accounted for in Table 2. In other words, balance training represents an essential part of the conditioning program at all stages of LTAD.

While RT on unstable surfaces is quite popular, Behm et al. (2015) concluded from their meta-analysis that the performance of unstable RT compared with stable RT has limited extra effects on muscular strength, power, and balance performance in healthy adolescents. Hence, the balance training exercises can be applied alone or in conjunction with the free weight RT.

Although there is not a single optimal training plan for all participants, a reasonable approach is to begin RT with one or two sets of 8–15 repetitions with a light to moderate load (30–60% 1 RM) with 8–12 exercises and a training frequency of at least two non-consecutive days per week (Behm et al., 2008). Youth with RT experience can progress to more intense or extensive training sessions (i.e., heavy resistance strength training) to achieve their training objectives (i.e., muscular strength, power, and endurance). In their meta-analysis, Lesinski et al. (2016) extracted dose-response relationships following RT in youth athletes for single RT parameters (e.g., training period, training frequency, training volume) independently and revealed that a training period of more than 23 weeks, five sets per exercise, 6 to 8 repetitions per set, a training intensity of 80–89% of the 1 RM, and 3–4 min rest between sets were most effective for conventional RT programs to improve muscular strength in youth athletes. Thus, it appears that heavy resistance strength training should be applied if the goal is to enhance muscular strength in young athletes (Table 2). However, these evidence-based findings should be adapted athlete specific considering individual abilities, skills, and goals (Lesinski et al., 2016).

The present review also demonstrates that for muscular power and athletic performance development, complex training involving RT and plyometric exercises should provide the largest magnitude of change. According to the Behm et al. (2008) review, improvements in athletic performance are typically only shown when RT was combined with specific sports training. Consequently, plyometric training should not be the only component of an exercise program, with the most desirable approach to incorporate other types of strength, conditioning and sport-specific practice into a well-rounded program (Behm et al., 2008). It is recommended that youth should start plyometric training with less-intense drills (e.g., double-leg jumps) and gradually progress to more advanced drills (e.g., single-leg hops) as balance, competence and confidence improves (Behm et al., 2008). Relatively few repetitions (i.e., < 10) are needed to bring about significant performance gains (Lephart et al., 2005). Plyometric training should occur on yielding surfaces (e.g., gymnasium floor or playing field) and the focus of early training should be on proper athletic positioning and landing (Behm et al., 2008).

While there is evidence that core strength training is effective in improving trunk muscle endurance (Granacher et al., 2014), it has limited effects on proxies of athletic performance in youth athletes (Prieske et al., 2016b). In fact, Prieske et al. (2016b) recently conducted a systematic review and meta-analysis on the effects of core strength training on proxies of trunk muscle fitness (e.g., time in plank test) and athletic performance (e.g., 5000-m run time) in trained individuals aged 16–44 years. The authors observed that irrespective of the athletes' expertise level (i.e., recreational, sub-elite, elite athletes), core strength training is an effective means to increase measures of trunk muscle fitness (ES = 1.07) but has no effects on proxies of athletic performance (ES = 0). Nevertheless, core strength training should be an integral part of conditioning programs during all stages of LTAD (Table 2) because it may enable youth athletes to withstand the demands of long-term athletic training and competition (Faigenbaum et al., 2016).

The exertion of eccentric training should be emphasized during later LTAD stages (i.e., training to train) to lower the risk of sustaining injuries in the muscle-tendon unit (Table 2). Of note, adolescence may be regarded as a critical phase of tissue plasticity in young growing athletes, as the adaptation process of the muscle-tendon unit is affected by both, environmental mechanical stimuli and maturational processes (Mersmann et al., 2014). In fact, non-uniform development of muscle strength and tendon mechanical and morphological properties have been found in adolescent athletes which increase the risk of sustaining overuse injuries (Mersmann et al., 2014). There is evidence that specific eccentric stimuli have a positive effect on the balanced development of muscle and tendon.

Table 2 contains a summary of the practical implications of this scoping review for coaches and fitness professionals as it illustrates a conceptual model containing adequate RT types for the application of conditioning programs during the stages of LTAD based on expert opinion and according to Lesinski et al. (2016), Faigenbaum et al. (2016), Lloyd et al. (2011, 2015), Balyi et al. (2013), and Kraemer and Fleck (2005). In this context, balance training appears to be an important preparatory (facilitating) training program during all stages of LTAD but particularly during the early stages. As youth athletes become more mature, specificity and intensity of RT methods increase. During the last years, increased research efforts have been accomplished to elucidate the effects of RT on muscular fitness and athletic performance in youth athletes. Nevertheless, with this scoping review we were able to identify research gaps in the literature that should be addressed in future studies (Table 3).

## Author contributions

All authors listed, have made substantial, direct and intellectual contribution to the work, and approved it for publication.

## Funding

This study is part of the research project “Resistance Training in Youth Athletes” (http://www.uni-potsdam.de/kraftprojekt/english.php) that was funded by the German Federal Institute of Sport Science (ZMVI1-081901 14-18).

### Conflict of interest statement

The authors declare that the research was conducted in the absence of any commercial or financial relationships that could be construed as a potential conflict of interest.
